# Homogentisate 1,2 Dioxygenase is Expressed in Human Osteoarticular Cells: Implications in Alkaptonuria

**DOI:** 10.1002/jcp.24018

**Published:** 2011-11-21

**Authors:** Marcella Laschi, Laura Tinti, Daniela Braconi, Lia Millucci, Lorenzo Ghezzi, Loredana Amato, Enrico Selvi, Adriano Spreafico, Giulia Bernardini, Annalisa Santucci

**Affiliations:** 1 Dipartimento di Biotecnologie, Università degli Studi di SienaSiena Italy; 2 Centro Interdipartimentale per lo Studio Biochimico delle Patologie Osteoarticolari, Università degli Studi di SienaSiena Italy; 3 Toscana Life Sciences FoundationSiena Italy

## Abstract

Alkaptonuria (AKU) results from defective homogentisate1,2-dioxygenase (HGD), causing degenerative arthropathy. The deposition of ochronotic pigment in joints is so far attributed to homogentisic acid produced by the liver, circulating in the blood and accumulating locally. Human normal and AKU osteoarticular cells were tested for *HGD* gene expression by RT-PCR, mono- and 2D-Western blotting. *HGD* gene expression was revealed in chondrocytes, synoviocytes, osteoblasts. Furthermore, HGD expression was confirmed by Western blotting, that also revealed the presence of five enzymatic molecular species. Our findings indicate that AKU osteoarticular cells produce the ochronotic pigment in loco and this may strongly contribute to induction of ochronotic arthropathy. J. Cell. Physiol. 227: 3254–3257, 2012. © 2011 Wiley Periodicals, Inc.

Alkaptonuria (AKU) results from a deficiency of homogentisate1,2-dioxygenase (HGD) that degrades homogentisic acid (HGA), an intermediary product of tyrosine catabolism.

In AKU, HGA oxidizes to benzoquinone acetic acid that forms melanin-like polymers that are progressively deposited in the connective tissue, most commonly joints, cardiovascular system, kidney and skin causing a pathologic pigmentation known as ochronosis. The most severe AKU symptom is degenerative arthropathy resulting from ochronosis in joint tissues, especially in shoulders, hips and knees. The intervertebral discs show generation and cause sciatica, lordosis, and kyphosis. Patients suffering from AKU-induced ochronosis experience considerable pain, incapacity, and disability. AKU still lacks of an appropriate therapy.

HGD is reported expressed in liver, kidney, prostate, small intestine, colon (http://www.uniprot.org/uniprot/Q93099). Ochronotic pigment deposition in joints has been so far attributed to HGA excess produced by the liver, circulating in the blood and eventually accumulating in the osteoarticular tissue.

We present results indicating that osteoarticular compartment cells express HGD and thus contribute to the production of local ochronosis in AKU arthropathy.

## Materials and Methods

### Human articular cells

Specimens were obtained after surgery for total hip replacement from three osteoarthritis and two AKU patients (range 58–70 age), defined according to the American College of Rheumatology criteria (Altman et al., 1991), with the Local Ethics Committee approval. Chondrocytes were obtained from femoral heads cartilage (Tinti et al., 2010). Osteoblasts and synoviocytes were isolated and cultured as described (Alvaro-Gracia et al., 1990; Gallagher et al., 1996).

### HGA treatment

First passage chondrocytes were seeded in 24-well plates at a starting density of 4 × 10^4^ cells/well until subconfluence. Cells were treated with 0.33 mM HGA in DMEM supplemented with 10% FCS (Braconi et al., 2010; Tinti et al., 2010, 2011b).

### Transmission electron microscopy

Samples were analyzed as described (Fioravanti et al., 2010).

### HGD expression

Chondrocytic RNA was extracted by RNaesy Plus Mini Kit (Qiagen, Milan, Italy). cDNA was synthesized by QuantiTect Reverse Transcription Kit (Qiagen). qPCR was designed for *HGD* using Porphobilinogen deaminase (HMBS) as reference gene for normalization. Primers were from Qiagen. qPCR reactions were done on LightCycler 1.0, Roche Molecular Biochemicals, Milan, Italy with LightCycler Software Version 3.5. qPCR amplification was performed in triple using QuantiFast SYBR Green PCR Kit (Qiagen). Reaction conditions were: 5′ at 95°C for HotStart polymerase activation, 40 cycles of 15 sec at 95°C and 30 sec a 60°C for denaturation and annealing/amplification and the temperature was raised from 70°C to 95°C at 0,1°C/step.*HGD* Cq values and efficiencies of primers were calculated with LinReg (Ramakers et al., 2003), converted into relative quantities and normalized (Pfaffl, 2001).

### Western blotting

Osteoarticular (Gallagher et al., 1996; Tinti et al., 2010) and osteosarcoma SaOS2 cells (Spreafico et al., 2006) were cultured as described. Liver cytosol was from male Wistar rats. Cell protein lysates were resolved by SDS–PAGE or 2D-PAGE (Spreafico et al., 2006; Braconi et al., 2010), electrotransferred onto nitrocellulose and probed with Anti-HGD antibody, followed by Anti-Rabbit HRP-conjugated antibody (all Sigma–Aldrich, Milan, Italy). Detection was obtained by ImmunoStarHRP (Bio-Rad, Segrate, Milan, Italy), images were acquired using ImageScannerIII (GE Healthcare, Milan, Italy), and analyzed by ImageQuantTL (GE). HGD bands optical densities were normalized against actin (Anti-β-Actin, Sigma–Aldrich).

### Statistical analysis

ANOVA tests were used for the comparison of data. Differences of *P* < 0.05 were considered significant.

## Results

**Fig. 1:**
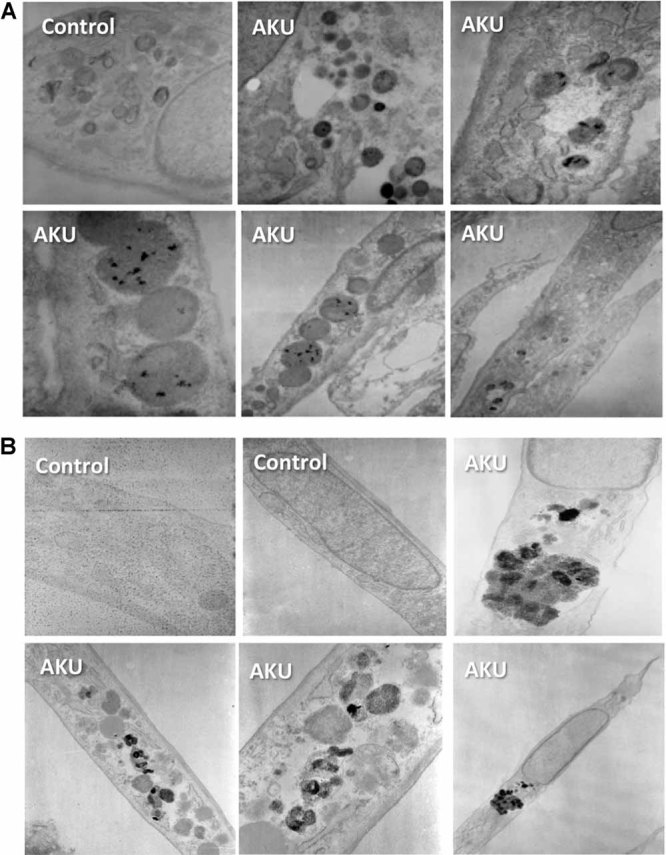
TEM micrographs of AKU chondrocytes (A) and synoviocytes (B). The images are representative of a cell population after analyzing 50–100 cells up to the fourth generation in each sample.

AKU chondrocytes contained ochronotic pigment (Fig. 1A). Observations of the pigment inside daughter cells until the fourth generation suggested that AKU chondrocytes were able to produce pigment. The same intracellular pigment was observable in ochronotic synoviocytes (Fig. 1B).

**Fig. 2:**
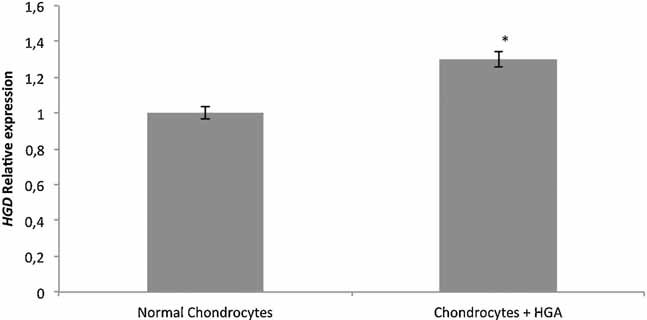
Expression of homogentisate 1,2 dioxygenase in human osteoarticular cells. Experiments were performed in triplicate. Bars represent mean ± SD. The asterisk indicates significant difference versus control (*P* < 0.05).

Expression of *HGD* gene was revealed in human chondrocytes (Fig. 2). Once treated with a HGA concentration within the range of that found in plasma of AKU patients, chondrocytes had a 1.3-fold change (30%) increase of *HGD* expression (*P* < 0.05).

**Fig. 3:**
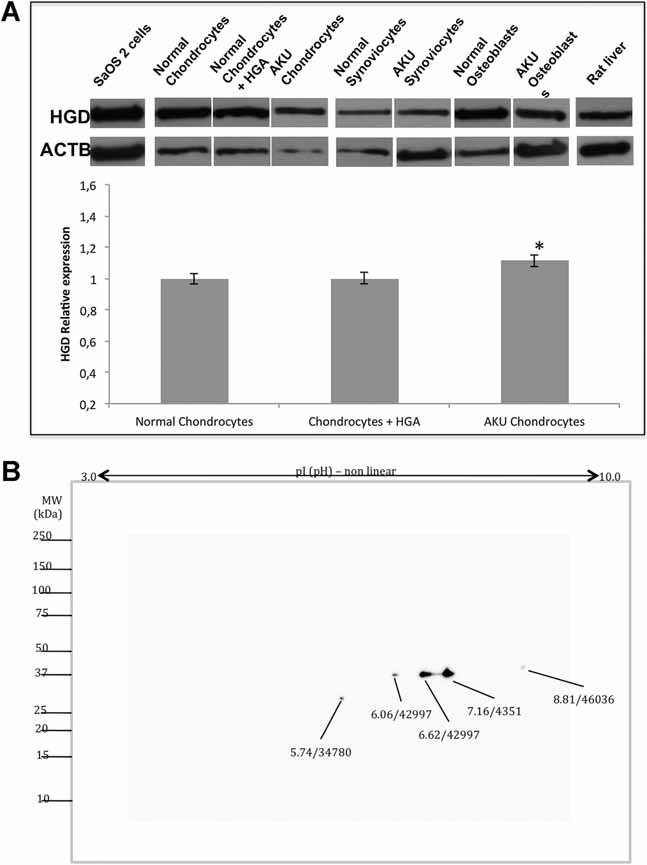
A: Western blotting with anti-HGD antibodies of human primary cultured chondrocytes. The use of rat liver as a positive control of human HGD presence was justified by the high level of homologyof the *HGD* immunogenic sequence between rat and human (94%, according to BLAST/UNIPROT algorithm). Experiments were performed in triplicate. Bars represent mean ± SD. The asterisk indicates significant difference versus control (*P* < 0.05). B: 2D Western blotting withanti-HGD antibodiesof human primary cultured chondrocytes. Mr and pI of the five HGD molecular species are reported.

Western blotting with anti-HGD antibodies confirmed the presence of HGD in human both normal and AKU chondrocytes, synoviocytes, osteoblasts as wells in human osteosarcoma cells (Fig. 3A). Treatment of normal chondrocytes with HGA did not alter HGD expression. On the contrary, in AKU chondrocytes a 10% increase of HGD expression was observed (*P* < 0.05), suggesting that a chronic condition of HGA excess may induce an enzyme overexpression.

2D-Western blotting confirmed HGD expression and revealed five different enzyme molecular species (Fig. 3B), ascribable to acetylation (http://www.uniprot.org/uniprot/Q93099) that neutralizes Lys positive charge, potentially changing protein function.

## Discussion

Osteoarticular tissue degeneration, chronic inflammation, and ochronotic arthropathy are the main severe AKU pathologic manifestations.

The current opinion is that osteoarticular cells do not express *HGD* and that AKU joint cells are innocent bystanders since pigments are produced elsewhere in the body, liver being the main organ involved. If this would be true, cultured cells, in the absence of body blood circulation, should not contain ochronotic pigment. On the contrary, this is the first report indicating that all the cells of the osteoarticular compartment express *HGD*, both under normal and AKU conditions. This implies a spontaneous ability of AKU osteoarticular cells to accumulate HGA and produce ochronotic pigment in situ, thus strongly contributing to induction of local ochronosis in AKU arthropathy.

The examination of AKU cartilage and of an ex vivo AKU cartilage model (Taylor et al., 2011, 2010; Tinti et al., 2011a), showed that HGA-induced pigmentation is non-homogeneous, differently from what expected if deposition was merely due to diffusion, with two different types (matrix and cytoplasmic) observed and a distinct pattern of binding of ochronotic pigment starting from lacunae. HGA has a high affinity for collagenous cartilages, but deposition also occurs at non-collagenous sites, suggesting that local tissue factors may promote ochronosis. This suggested that pigment deposition is actually cell-mediated and possibly due to: (i) an active accumulation occurring independently in the lacunae based on different activation and maturation of cells present in the microenvironment, in agreement with data here presented and previous observations (Taylor et al., 2011, 2010; Tinti et al., 2011a); or (ii) a preferential deposition in pericellular areas due to pigment's affinity for some matrix structures.

Our findings about a local production of pigment may explain why, although cartilage and synovia are poorly vascularized, ochronosis is relevant in such type of tissue and why arthropathy is always severe in AKU. The observed increase of HGD expression in AKU cells is probably due to a compensatory mechanism to overcome the almost null catalytic activity of the deficient enzyme.

In situ production of ochronosis may have important implications for the treatment of AKU, since local therapies aimed to ameliorate ochronotic arthropathy may be envisaged.
